# Filling Root Canals of Human Teeth

**Published:** 1889-11

**Authors:** Isaac Douglas

**Affiliations:** Romeo, Mich.


ARTICLE VI.

### FILLING ROOT CANALS OF HUMAN TEETH.

BY ISAAC DOUGLAS, ROMEO, MICH.

[Read before the Michigan State Dental Society, June, 1889.]

It is not the purpose of this paper to introduce anything new and untried, nor to discuss at length the various modes and materials which have been long in use; but rather to detail the method that has given the most satisfactory results in an experience of over 37 years in one place, with ample opportunity to observe the workings of other methods that have been recommended by others and tried from time to time. It is hoped that at least one of the younger members of the profession may thus be spared the tasks of numerous experiments, and that the time thereby saved may be devoted to valuable discovery in new fields.

How shall we get into the root canal with the least injury to the tooth, and the greatest ease to patient and operator? This is a question of no little importance, and of some difficulty to decide. The first step toward becoming expert in filling the roots of teeth, is the careful study of the form of the dental pulp, particularly that part about the neck of the molars. This can best be done by a careful and thorough examination of those teeth that have been consigned to the forceps ; not merely one tooth from the various portions of the dental arch, but many teeth from each location posterior to the bicuspids, paying particular attention to the peculiar form and size of the canals well down the roots.

It is a comparatively easy matter to gain access to the root of a bicuspid ; bearing in mind that the first upper bicuspids most frequently have two root canals, while the other bicuspids rarely have more than one. This single canal, however, sometimes bifurcates at the neck, being united one-fourth or one-half way down.

It is useless to attempt to fill the roots of any tooth posterior to the cuspids, without having free and direct access to such root canal in a line with its entrance into the root.

A drill should never be used to enlarge or alter the form of a root canal. Yet it is occasionally necessary to use a drill to remove a pulp pebble that extends far into the posterior root of a lower molar, or a palatine root of an upper molar, and, possibly, in a bicuspid. But it is rarely advisable to use a drill at all as it leaves the canal in an ill shape for further procedure; the drill is almost sure to leave corners, or at least a roughness, which more or less interferes with the ready introduction of instruments or materials for filling. In the natural state the canals are smooth throughout their entire length. They may be curved or tortuous, but they can so rarely be improved by the use of drills, that I have destroyed a part of my root canal drills, lest I might be tempted to use them.

To obtain the best entrance to the root canal of the anterior six upper teeth, requires careful consideration. The success of the operation must be secured, yet the strength, durability aad beauty of the tooth must not be impaired. Perhaps there is a good filling in each approximate side of an incisor or a bicuspid, and these fillings may extend so far toward the palatine center, that, to drill another opening would too much weaken that side of the tooth. In such a case, that filling should be removed which would give the most desirable entrance to the canal. Other things being equal, in case of a lateral incisor or a cuspid, it is better to enter from the anterior approximate side; because if one of these roots should be curved, which is very liable to be the case, we would thus gain a more direct access to its apex, particularly if the incising end of the crown is intact. The drill should then start at a point about one-thirty-second or one-sixty-fourth of an inch from the outer surface of the tooth near the lingual portion of the cavity; the drill should be held at an angle of twenty or thirty degrees to the root canal, so as to reach it about one-half or one-third the way up the foot. Care must be taken not to leave too much of an angle between the root canal and the one we have just made; also, to avoid letting the drill touch the opposite side and make a depression that will cause annoyance subsequently.

The first tool to introduce into a root canal is a broach, and this should be the best five-sided jeweler's watch broach. By proper annealing, a broach may be made perfectly soft, or spring tempered, according to the work it is required to do.

To obtain a spring-temper, take a piece of Russia iron one-half inch wide and four to six inches long, fold it lengthwise nearly upon itself, making a trough; by putting one or more broaches into this trough and holding it in the flame of a lamp, any desired degree of temper may be obtained.

To make broaches full soft, take a very small metal tube, put the broaches into it and close both ends; heat to a cherry red and let it cool before opening the tube. The toughness of these broaches may be tested by bending or twisting them into any desired form. With a sharp chisel or a well tempered knife, cut fine, short barbs on the broach from its point back one-eighth or one-fourth of an inch. We now have for 35 cents per dozen, a much better article than can be obtained at a dental depot for 75 cents.

With a broach prepared in this way, enter nearly or quite to the apex of the root. The pulp is supposed to be dead, as the treatment of the live pulp is a topic outside of the present paper. After giving it from one to three turns, according to the amount of resistance, withdraw the broach, when, if the pulp has been carefully cut off at the juncture of the drill hole and the canal, in case of recent death of the pulp, that portion of the pulp which is nearest the apex will have been brought out. If the pulp has been dead a sufficient length of time to have separation complete, there will be no hemorrhage.

The next article to us, we will call, for want of a better name, a paper point. It is made in the following manner: from a sheet of bibulous paper, not Japanese, cut a strip three-fourths of an inch wide; from this strip cut at such an angle as to make three-cornered pieces one or one and one-fourth inches long. Fold the nearest corners together, doubling the strip its entire length; then roll and twist between the thumb and finger until it is round and hard its entire length.

There are many things that may be said in favor of these paper points. They are not very apt to push any particles of septic matter through the foramen. Any loose septic substance adheres to them so readily, that they assist greatly in cleaning the canal. They may be turned in the canal, and, being so similar in taper, wipe the sides without forcing the debris toward the apex of the root. They will enter the finest root canal as far as a broach can be passed.

With one of these paper points, enter the root canal to the apex, let rest a few moments to drink up the pus globules, remove and introduce another, and so on until they come out dry; then dip one in carbolic acid or creosote, introduce and let it rest; while, with a pair of slender pointed gold carriers, we take up a part of a drop of the same fluid and carry to the first end of the drill hole, carefully separate the points of the tweezers, and the fluid will run directly into the canal by capillary attraction. After another thorough drying with paper points, this part of the canal is ready for filling; and the permanent filling for this part of the canal should invariably be done at the same sitting, even if no other portion of the root canal is filled at this time. After this is done, proceed to open into the rest of the canal and the pulp chamber, and prepare the same for filling. This had been carefully omitted up to this time, lest chips of the dentine get into the canal and cause annoyance.

Suppose another condition—a case in which the pulp has been dead a long time, no indications of abscess having occurred. One very important object is to avoid forcing any septic substance through the apical foramen, which is liable to be enlarged. The broach should be introduced very cautiously, part way at a time, and removed, until the apex is reached; taking care not to go through, so as to cause much disturbance of the soft tissues. A slight hemorrhage by a single prick of the broach does no harm, and may be beneficial by aiding the removal of any particles of septic matter that may be in the apical foramen or slightly beyond it. The same may be done by $H_2O_2$, but the method here given is quite as successful without its use.

After removing as much septic substance as possible with the broach, use one or two paper points to dry the canal, if there is much moisture present; then introduce carbolic acid or creosote as before described, and dry as many times as is necessary to have the paper come out clean. In case the paper points reach through the apex so as to cause pain, indicated by a more or less perceptible tremor of the face, these points should be broken off leaving them large enough to prevent their passing the foramen; this will be of utility as we proceed.

One case more. If the pulp has been dead for some time and suppuration has commenced but quite recently, on opening the pulp chamber and removing the putrescent pulp, the pain is relieved by its discharging pus through the tooth for a few minutes. When the perceptible discharge ceases, dry the canal as before described, wash out and dry again and again, until there is no more discharge and the soreness is gone. The root may then be prepared and filled at once. Give the patient a few doses of mercurious vivus or solubillis 3x, and there will be no further trouble with the tooth.

I believe I was the first to introduce root canal pluggers made of piano-wire, using pen-holders for handles. Several years afterward I ordered some steel handles made by our much lamented S. S. White, who soon offered the same kind of handles for sale, illustrated in the *Cosmos*.

Pluggers for this purpose should be spring-tempered, and yet soft enough to be bent and re-bent a great number of times; and this wire possesses the necessary qualities in a remarkable degree. These may be filed down to any desired size or shape, but the less they are filed the tougher they remain. Instead of filing much, use different sizes of wire. A little experience will enable one to put a very good point on them. Take hold of the wire after it is filed to the desired size, with a pair of flat-nosed pliers, and bend a little back and forth until it breaks off. Sharpen the other end of the wire three-cornered, and drill the wood slightly, if hard, grasp the wire with strong pliers and drive into the wood. The wood handles answer quite as well as steel.

Armed with several sizes of these pluggers, proceed to prepare the filling. Of all the materials that have been tried and recommended for this purpose during the last third of a century, none, in our hands, gives such perfect satisfaction as gold. This may be prepared so as to make a bad filling; or, it may be prepared so as to make the best possible filling in all ordinary cases. It requires years of experimenting to learn the best method of preparing and using gold for this purpose; but, when prepared and used in the right manner, it will fill the finest root canal as far as the broach can be passed, and yet it need seldom, if ever, be passed through the foramen.

The gold should be prepared in the following manner: tear the gold into irregularly shaped pieces, from one-half to three-fourths of an inch wide, and from three-fourths to one and one-fourth inches long; lay it upon the palm of the hand, lengthwise of the wrinkle caused by closing the hand; lay a long, straight, fine plugger on the gold lengthwise the wrinkle; half flex the fingers, keeping the fingers as straight as possible; withdraw the plugger, keeping the hand flexed; strike the plugger down into the fold of the hand repeatedly, until all of the gold is carried into the bottom of the fold of the hand. Now open the hand, lay the index finger lengthwise the gold and roll it (the gold) to and fro, forming the gold round and slender. Prepare many of these, varying in size. Select one as fine as the finest broach for the finest root canal; take it in the gold tweezers and carry it as far into the canal as the broach has been. If the canal is a large one, or if the foramen is enlarged, select a larger roll of gold. As the gold has not been twisted, it is easily upset, and there is no occasion to push it through the foramen, provided we have ascertained the size of the foramen, and made the roll of gold too large to pass through.

There are various modes of ascertaining the size of an enlarged foramen, of which the following is the best that has not been mentioned: Pass a bur through, that will size it; follow it with a larger one, nearly through; measure accurately. A ball of gold, rolled hard to the size of the last bur, is placed as far up as the last bur went; the rest of the operation is simple.

Other methods of ascertaining the size of the foramen, use soft wood, a broach wound with cotton, etc.

Should the gold be forced through a little, it is as harmless as any insoluble substance. In a number of cases it has been worn thus projecting, for years without discomfort. Again, in using gold, there is no necessity of forcing air or anything else that is harmful through the foramen.

There is a satisfaction in feeling, as we proceed with the operation, that it is being perfectly performed, and that, when completed, it will be a success.—*Ohio Journal of Dental Science.*

